# The emergence of the hypervirulent *Klebsiella pneumoniae* (hvKp) strains among circulating clonal complex 147 (CC147) harbouring *bla*_NDM/OXA-48_ carbapenemases in a tertiary care center of Iran

**DOI:** 10.1186/s12941-020-00349-z

**Published:** 2020-03-31

**Authors:** Omid Pajand, Narges Darabi, Maedeh Arab, Raheb Ghorbani, Zakaria Bameri, Ali Ebrahimi, Zoya Hojabri

**Affiliations:** 1 Microbiology Department, Faculty of Medicine, Semnan University of Medical Sciences, Semnan, Iran; 2 Student Research Committee, Faculty of Medicine, Semnan University of Medical Sciences, Semnan, Iran; 3 Social Determinants of Health Research Center, Semnan University of Medical Sciences, Semnan, Iran; 4 Infectious Disease and Tropical Medicine Research Center, Zahedan University of Medical Sciences, Zahedan, Iran

**Keywords:** hvKp, NDM, OXA-48, *rmtC*, Phylogroup, Siderophore, Capsular type

## Abstract

**Background:**

*Klebsiella pneumoniae* is a public health concern because of its ability to develop multidrug resistance and hypervirulent genotypes, of those capsular types K1 and K2 cause community and nosocomial life-threatening infections. This study aimed to determine the antibiotic susceptibility patterns and genotypic traits of a collection of *Klebsiella* spp. isolates. Furthermore, the clonal relatedness of *bla*_NDM_ producing strains was investigated.

**Methods:**

During a 19-months  surveillance study, 122 *Klebsiella* spp. isolates were cultured from extraintestinal specimens of patients admitted to the tertiary referral hospital in Semnan, Iran. Isolates were identified using biochemical tests and subjected to determination of phylogroups, capsular types and virulence/resistance genes content. Hypervirulent *K. pneumoniae* (hvKp) strains were detected genotypically, and Enterobacterial Repetitive Intergenic Consensus (ERIC)-PCR fingerprinting was used to determine the clonality of *bla*_NDM_ producing strains.

**Results:**

Multidrug resistant phenotype was detected in 75 (61.5%) isolates and amikacin was found as the most potent antibiotic with the susceptibility rate of 85.2%. The carbapenemase genes were detected in 45 (36.8%) strains, including 21 (17.2%) *bla*_OXA-48_, 7 (5.6%) *bla*_NDM-1_, 14 (11.4%) *bla*_NDM-1/OXA-48_ and 3 (2.4%) *bla*_IMP-_ carrying strains, while 55 (45.08%) isolates showed carbapenem resistant phenotype. The first *bla*_NDM-1_ carrying strain was cultured from a sputum specimen on March 2015, while the last positive one was recovered from blood culture on September 2016. Most of the isolates (80.3%) belonged to phylogroup I, and *bla*_NDM-1_ was identified among all three phylogroups. The ERIC-PCR clustered the 101 *bla*_NDM_ negative and 21 *bla*_NDM-1_ positive isolates into 25 and five clusters, respectively, and the latter group belonged to clonal complex 147 (CC147). One K1 and 15 K2 *bla*_NDM-1_ negative isolates were detected, of those three strains were identified as hvKp. Five K2 positive strains, including four *bla*_OXA-48_ producer and one hvKp sequence type 86 (ST86) were carbapenem resistant. Among carbapenem resistant isolates, CC147 strains harboured higher rates of siderophores *iutA* and *ybtS*.

**Conclusion:**

The present findings showed a hospital circulation of CC147 *bla*_NDM-1_ or *bla*_NDM-1/OXA-48_ producing strains, disseminated in different wards. The hvKp/ST86 strain expressing K2 capsular type and carbapenem resistant phenotype wasn’t reported from Iran so far. So, it seems that we must be aware of the emergence and spread of new *K. pneumoniae* clones associated with resistant and hypermucoviscous phenotypes.

## Background

*Klebsiella pneumoniae*, one of the most important members of the *Enterobacteriaceae* family, is the leading cause of both of the community and healthcare-associated infections. The capsule, as a critical virulence factor in this organism, plays an important role in its pathogenesis and avoids phagocytosis [1]. Over the past decade, the emergence of hypervirulent variants of *K. pneumoniae (*hvKp) which characteristically express hypermucoviscosity phenotype caused serious concerns [2]. These strains carry virulence genes associated with invasive disease and may cause severe infections such as pyogenic liver abscesses and endophthalmitis, in immunocompetent, healthy individuals [2]. While the hvKp strains were rarely resistant to commonly used antibiotics when firstly described, the emergence of multidrug resistant (MDR) phenotypes among these strains are increasingly reported in recent years due to the dissemination of mobile genetic elements encoding drug resistance [2]. Of special concern is the acquisition of carbapenem resistance genes and development of carbapenem resistant phenotype, since these agents are the last resort of antibiotics for treatment of infections caused by multidrug resistant organisms [2].

Increasing recognition of carbapenemase producing *K. pneumoniae*, including class A (KPC), class B (IMP, VIM and NDM) and class D (OXA-48-like enzymes) carbapenemases has led to international concern, as they are carried on transposable elements in association with other resistance determinants, such as Extended spectrum β-lactamases (ESBLs), *ampC* cephalosporinases and 16S rRNA methyltransferases [3]. Indeed, the convergence of carbapenemase production and hypermucoviscosity phenotype in this organism poses an important threat to public health.

The *bla*_NDM_, a relatively newly described Metallo-β-lactamase (MBL), was first identified in *K. pneumoniae* and *E. coli* isolated from a Swedish patient who was hospitalized in India in 2008 [4]. Since then, it has spread worldwide and now NDM producing Gram-negative bacilli have been reported from more than 40 countries, and the Indian subcontinent and the Middle East are considered as the main reservoirs for *bla*_NDM_ producing bacteria [5]. Iran, as one of the Middle East countries, neighbor countries where the NDM and OXA-48 producing bacteria are endemic [6]. While *bla*_NDM_ producing *K. pneumonia*e isolates have been reported from different cities of Iran in recent years, sequence types of these isolates are determined and published from three cities located in the center, south, and south-east of this country [6–8]. In our previous study, we reported a relatively high prevalence of *bla*_OXA-48_/*bla*_NDM-1_ producing *Enterobacteriaceae* isolates collected from the large tertiary hospital of Semnan [9], an important city along the historical Silk Road. So in this survey, our goal was first to determine the phylogenetic groups, capsular genotypes, hypermucoviscosity biomarkers, and resistance determinants of *K. pneumoniae* isolates collected during 19-months surveillance study and second, to carry out sequence typing of representatives of NDM producing isolates based on the Enterobacterial Repetitive Intergenic Consensus (ERIC) fingerprinting.

## Methods

### Sample collection

A 19-months surveillance study was conducted in the main tertiary teaching hospital of Semnan, Iran (Kosar hospital). During March 2015 to September 2016, 122 non-duplicate *K. pneumoniae* isolates were recovered from clinical specimens of patients admitted to the hospital. The isolates were cultured from different extraintestinal specimens including urine, wound, sputum, blood and tracheal aspirate. Specimens were collected as the routine diagnostic purposes. *K. pneumoniae* isolates were identified based on the biochemical reactions, including reaction on Triple Sugar Iron (TSI) agar, SH2/Indole/Motility (SIM) pattern, growth on Simmon-citrate agar medium, urease production on urea agar, Methyl Red/Vogues Proskauer (MR/VP), and Ornithine decarboxylase (OD) test. Isolates were confirmed by PCR in which both *k. pneumoniae* subsp. *Pneumoniae* and subsp. *ozaenae* give a 130 bp band, and subsp. *rhinoscleromatis* is negative [10]. *K. oxytoca* species were identified based on the VP +/Indole +/OD negative, tests results [11].

### Antimicrobial susceptibility testing

Antibiotic susceptibility patterns for 16 antibiotics were obtained using standard disc diffusion test. For carbapenem non-susceptible isolates (resistant to either of the imipenem, meropenem, and ertapenem) carbapenem MICs were determined using gradient E-test strips (Liofilchem, Italy). Susceptibility testing results were interpreted according to the Clinical and Laboratory Science Institute (CLSI) recommendations. The halo zones of ≥ 23 mm and ≥ 22 mm were considered as susceptible breakpoints for imipenem/meropenem and ertapenem, respectively [12]. Multidrug-resistant (MDR) isolates were those resistant to at least one representative of ≥ 3 antimicrobial classes—that is, penicillins (ampicillin/sulbactam, piperacillin-tazobactam), Extended Spectrum Cephalosporins (ESCs)/monobactams (cefotaxime, ceftazidime, cefepime/aztreonam), aminoglycosides (gentamicin), fluoroquinolones (ciprofloxacin), and antifolate agents (trimethoprim-sulfamethoxazole) [13]. Extended spectrum β-lactamase (ESBL) producing strains were identified using phenotypic combined disk test as recommended by CLSI [12].

### Detection of resistancegenes and capsular typing

Genomic DNA of collected isolates was extracted using Cetyl trimethylammonium bromide (CTAB) method [14, 15]. The isolates carrying carbapenemases (*bla*_NDM-_, *bla*_IMP-_, *bla*_VIM-_, *bla*_OXA-48_, *bla*_KPC_) [16], extended spectrum β-lactamases (*bla*_TEM-_, *bla*_SHV-_, *bla*_OXA-1_, and CTX-M clusters including CTX-M-G1, G2, G8, G9 and G25) [16], plasmid mediated quinolone resistance (PMQR) (*qnrA*, *qnrB*, *qnrS*, *aac*-*6Ib*-*cr*) [17], aminoglycoside resistance determinants (ARD) *aac*-*6Ib*, *aac3IIa* and 16S rRNA methyltrasfrases (*armA*, *rmtB*, *rmtC*) were detected as described previously [16]. The capsular genotypes, including K1 (using magA primers), K2, K5, K20, K54 and K57, which are strongly associated with invasive disease, were determined by multiplex PCR as described previously [10]. The presence of plasmid-encoding virulence genes, including *iucA (*aerobactin siderophore biosynthesis), the plasmid-borne *rmpA* gene (*prmpA*), *prmpA2*, (regulators of the mucoid phenotype via increased capsule production), and *peg*-*344 (*putative transporter) which have been shown experimentally to contribute to hypervirulence in in vivo infection models, was assessed as described by Russo et al. [18]. Of the virulence genes which are associated epidemiologically with putative hvKp strains, carriage of the *iroB (*salmochelin siderophore biosynthesis), *iutA* (receptor for hydroxamate siderophore), *allS* (associated with allantoin metabolism), *mrkD* (type 3 fimbrial adhesion) and *ybtS* (yersiniabactin) genes was investigated by multiplex PCR [18, 19]. Furthermore, the K2 positive strains were subjected to a multiplex PCR to identify the main hvKp clones, including clonal group (CG) 380, sequence type (ST) ST86, ST65 and ST375, as described by Davenet et al. [20].

### Phylogenetic determination and clonal relatedness

For phylogenetic analysis, *gyrA* PCR–RFLP using restriction enzymes *TaqI* and *HaeIII* was performed as described by Brisse et al. [21]. The clonality of strains was determined by ERIC-PCR fingerprinting and the obtained dendograms were analyzed with Bionumerics software, version 6.1 (Applied Maths, Sint-Martens-Laten, Belgium). The similarities in amplicon profiles were compared using a Dice coefficient at 1% tolerance and 0.5% optimization, and a dendogram was constructed using the unweighted-pair group method, with a cutoff of 80% similarity [22]. Sequence type of isolates was determined for representatives of NDM positive strains of each cluster of ERIC dendogram according to the *K. pneumoniae* MLST website (https://bigsdb.pasteur.fr/klebsiella/klebsiella.html).

### Statistical analysis

Dichotomous variables were described using frequencies and percentages, and they were compared using Chi square test, as appropriate. The criterion for statistical significance was *P *< 0.05. Data were analyzed with software SPSS version 16.

## Results

### Patients and isolates

In this study, a total of 122 *Klebsiella* spp. isolates were recovered from all clinical specimens except for stool. Most of the isolates were cultured from urine (80, 65%), followed by sputum (33, 26.8%), wound (5, 4.1%), blood (2, 1.6%) and chest tube (1, 0.8%). Four strains, including two *Klebsiella* subsp. *ozaenae* (one cultured from urine, other one from sputum), one *Klebsiella* subsp. *rhinoscleromatis (*cultured from urine) and one *Klebsiella oxytoca (*cultured from urine) were identified among collected isolates.

### Antibiotic susceptibility patterns and frequency of resistance genes

The highest susceptibility rate was obtained against amikacin (104, 85.2%), followed by meropenem (81, 66.4%), imipenem (77, 63.1%) and gentamicin (74, 60.7%). The presence of *bla*_OXA-48_, *bla*_NDM-_, both of *bla*_OXA-48_ and *bla*_NDM-_ (*bla*_OXA-48/NDM_) and *bla*_IMP-_ genes was detected among 21 (17%), 7 (5.6%), 14 (11.3%) and 3 (2.4%) of isolates, respectively. The NDM PCR products from all positive strains were subjected to sequencing and identified as NDM-1 variant [9]. The most active agents against *bla*_NDM-1_ producers were amikacin (13, 61.9%) and trimethoprim/sulfamethoxazole (3, 14.3%) (Table 1).

**Table 1:** Antibiotic resistance rates and frequency of resistance genes among NDM positive and negative isolates

	NDM positive (n = 21)	NDM negative (n = 101)	NDM positive vs. NDM negative *P* value
Resistance rates n (%)			
Imipenem	21 (100)	24 (23.8%)	*0.001*
Meropenem	21 (100)	20 (19.8%)	*0.001*
Ertapenem	21 (100)	30 (29.7%)	*0.001*
Cefepime	20 (95.2)	39 (38.6%)	*0.001*
Ceftazidime	21 (100)	52 (51.5%)	*0.001*
Cefotaxime	21 (100)	66 (65.3%)	*0.001*
Aztreonam	21 (100)	51 (50.5%)	*0.001*
Ampicillin/sulbactam	21 (100)	54 (53.5%)	*0.001*
Piperacillin/tazobactam	21 (100)	36 (35.3%)	*0.001*
Amoxicillin/clavulanate	21 (100)	53 (52.5%)	*0.001*
Ciprofloxacin	20 (95.2)	48 (47.5%)	*0.001*
Levofloxacin	20 (95.2)	37 (36.6%)	*0.001*
Gentamicin	20 (95.2)	28 (27.7%)	*0.001*
Tobramycin	20 (95.2)	33 (32.7%)	*0.001*
Amikacin	8 (38.1)	10 (9.9%)	*0.003*
Trimethoprim/sulphamethoxazole	18 (85.7)	46 (45.5%)	*0.001*
MDR	21 (100)	54 (53.5)	*0.001*
Presence of resistance and capsular markers n (%)			
*armA*	10 (47.6)	16 (15.8%)	*0.003*
*rmtC*	3 (14.3)	3 (3%)	0.06*
*aac6Ib*	14 (66.7)	35 (34.7%)	*0.01*
*aac3IIa*	19 (90.5)	28 (27.5%)	*0.001*
*qnrB*	1 (4.8)	22 (21.8%)	0.1
*qnrS*	18 (85.7)	17 (16.8%)	*0.001*
*aac6Ib*-*cr*	19 (90.5)	47 (47%)	*0.001*
*bla*_OXA-48_	14 (66.7)	21 (20.8%)	*0.001*
*bla*_IMP-_	0	3 (3%)	1
CTX-M-G1	21 (100)	53 (52.5%)	*0.001*
CTX-M-G2	0	3 (3%)	0.5
CTX-M-G8	0	2 (2.0%)	0.6
CTX-M-G9	0	2 (2.0%)	0.6
CTX-M-G25	0	7 (7%)	0.2
*bla*_TEM-_	12 (57.1)	29 (28.7%)	*0.02*
*bla*_SHV-_	19 (90.5)	67 (66.3%)	*0.03*
*bla*_OXA-1_	15 (71.4)	25 (24.8)	*0.001*
K1	0	1 (1%)	1
K2	0	15 (14.8)	0.07

Italic values indicate the significance of *P* value (*P* < 0.05)

*MDR* multidrug resistance

*P value was borderline

**Fig. 1:**
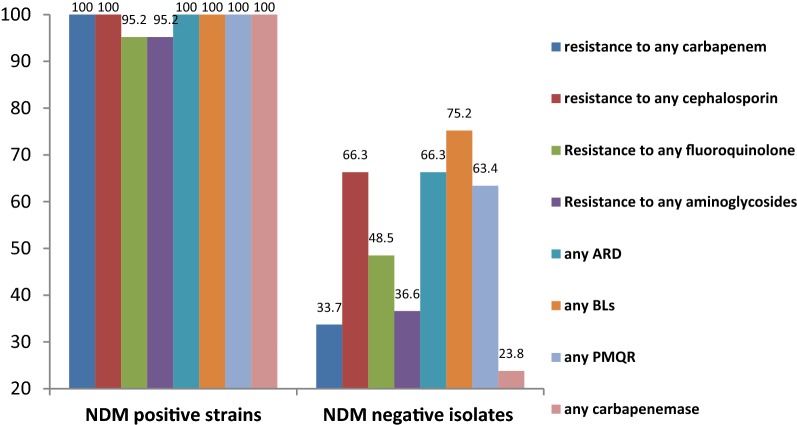
Frequency (percent) of any resistance determinants, and resistant phenotype to any carbapenems, any cephalosporins, fluoroquinolones and any aminoglycosides in two groups of NDM positive and negative isolates. Any carbapenemase: *bla*_OXA-48_, *bla*_NDM-1_, *bla*_IMP-_; any PMQR: *qnrB*, *qnrS, aac6Ib*-*cr*; any β-lactamase (BL): *bla*_TEM-_, *bla*_SHV-_, CTX-M, *bla*_OXA-1_; any ARD: *aac3IIa*, *aac6Ib*, *armA*, *rmtC*

One of the 21 *bla*_NDM-1_ producing isolates was identified as subsp. *ozaenae* (isolate no. 500A, cultured from sputum). The *bla*_NDM-1_ producers were mostly cultured from urine (10, 47.5%), followed by sputum (9, 42.9%), wound and blood (one, 4.8% for each of them). Carbapenemase encoding gene, including either of the *bla*_OXA-48_, *bla*_NDM-1_, or *bla*_IMP-_was detected in 43 strains out of 55 carbapenem resistant (resistant to at least one of the three carbapenems) isolates (78.2%, *P *< 0.001), and the two *bla*_IMP-_ carrying strains were found susceptible to carbapenems. All of the *bla*_NDM-1_ carrying strains were found non-susceptible to carbapenems, with the MICs ranging from 1.5 to > 32 µg/ml [9]. As compared to *bla*_NDM_ negative isolates, *bla*_NDM-1_ carrying strains showed significantly higher carriage rates of any resistance determinants (PMQR, ARD or β-lactamases) and higher resistance rates against any studied antibiotics (*P *< 0.001) (Fig. 1). Of the ARD studied, the presence of *armA* (*P:* 0.01), *aac6Ib* (*P *< 0.001), and *aac3IIa* (*P *< 0.001) was associated with resistant phenotype to any of the aminoglycoside antibiotics. Of the PMQR determinants, the carriage of *qnrS* (*P *< 0.001) and/or *aac6*-*Ib*-*cr* (*P*: 0.03) genes was associated with resistance to any of the fluoroquinolones. For β-lactamase genes, isolates harbouring the *bla*_TEM-_, *bla*_SHV-_, *bla*_OXA-1_ and/or CTX-M-G1 were resistant to any of the three studied cephalosporins (*P *< 0.001).

The resistance determinants which detected significantly among NDM producers were *bla*_OXA-48_, *bla*_TEM-,_*bla*_SHV-_, *bla*_OXA-1_, *armA*, *aac3IIa*, *aac*-*6Ib*, *aac6*-*Ib*-*cr*, *qnrS*, and CTX-M-G1. The co-carriage of *rmtC* and *bla*_NDM-1_ was not found statistically significant, however this association was on the border of significance (*P*: 0.06).

### Genetic relatedness and sequence typing of *bla*_NDM_ producers

**Fig. 2:**
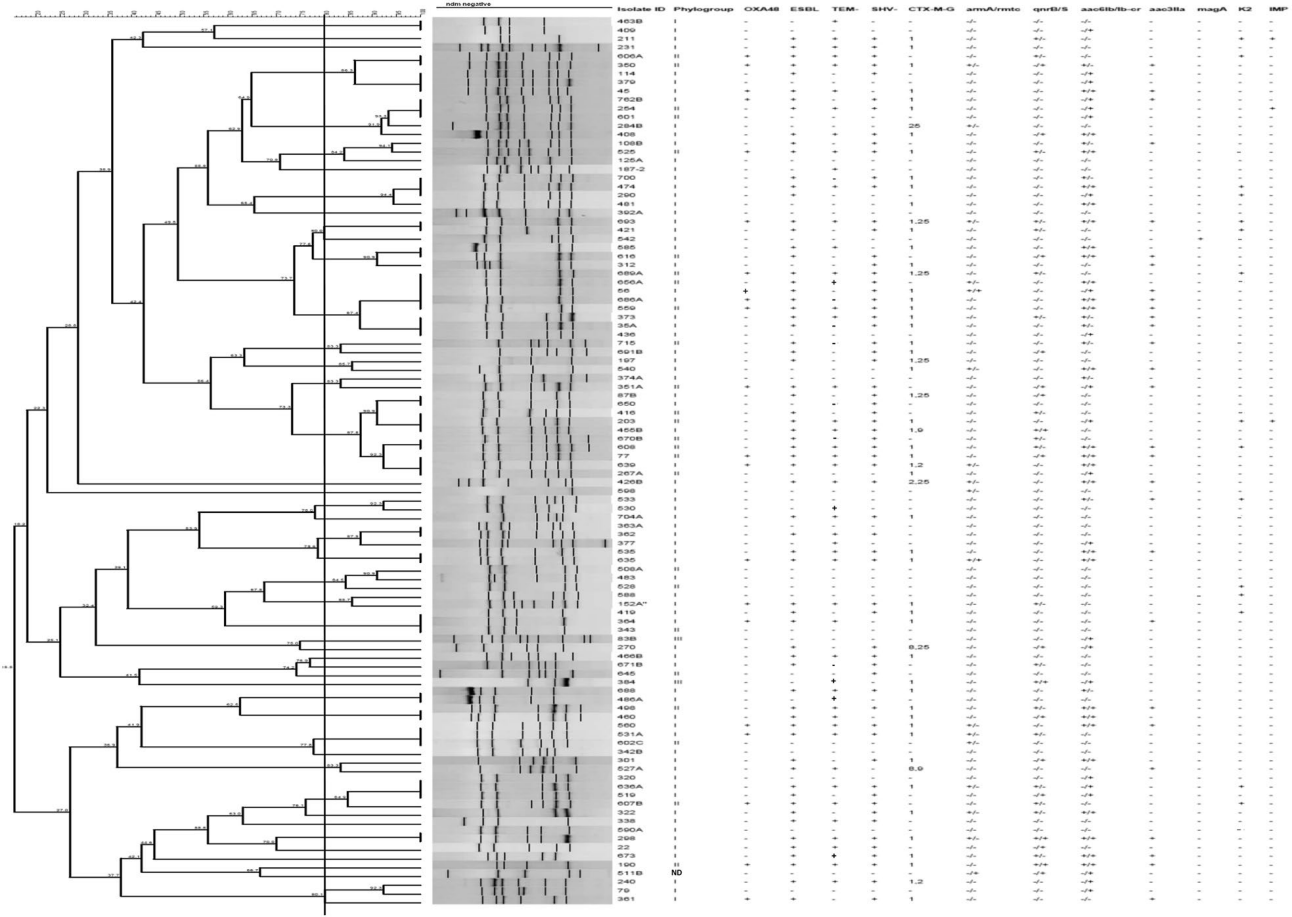
ERIC-PCR based dendogram of *bla*_NDM_ negative isolates. Cluster analysis of the Dice similarity indices based on the unweighted pair group method using average linkages (UPGMA) was done to generate a dendogram describing the relationship among the ERIC profiles. ESBL: indicate the phenotypic results, IMP: imipenemase

**Fig. 3:**
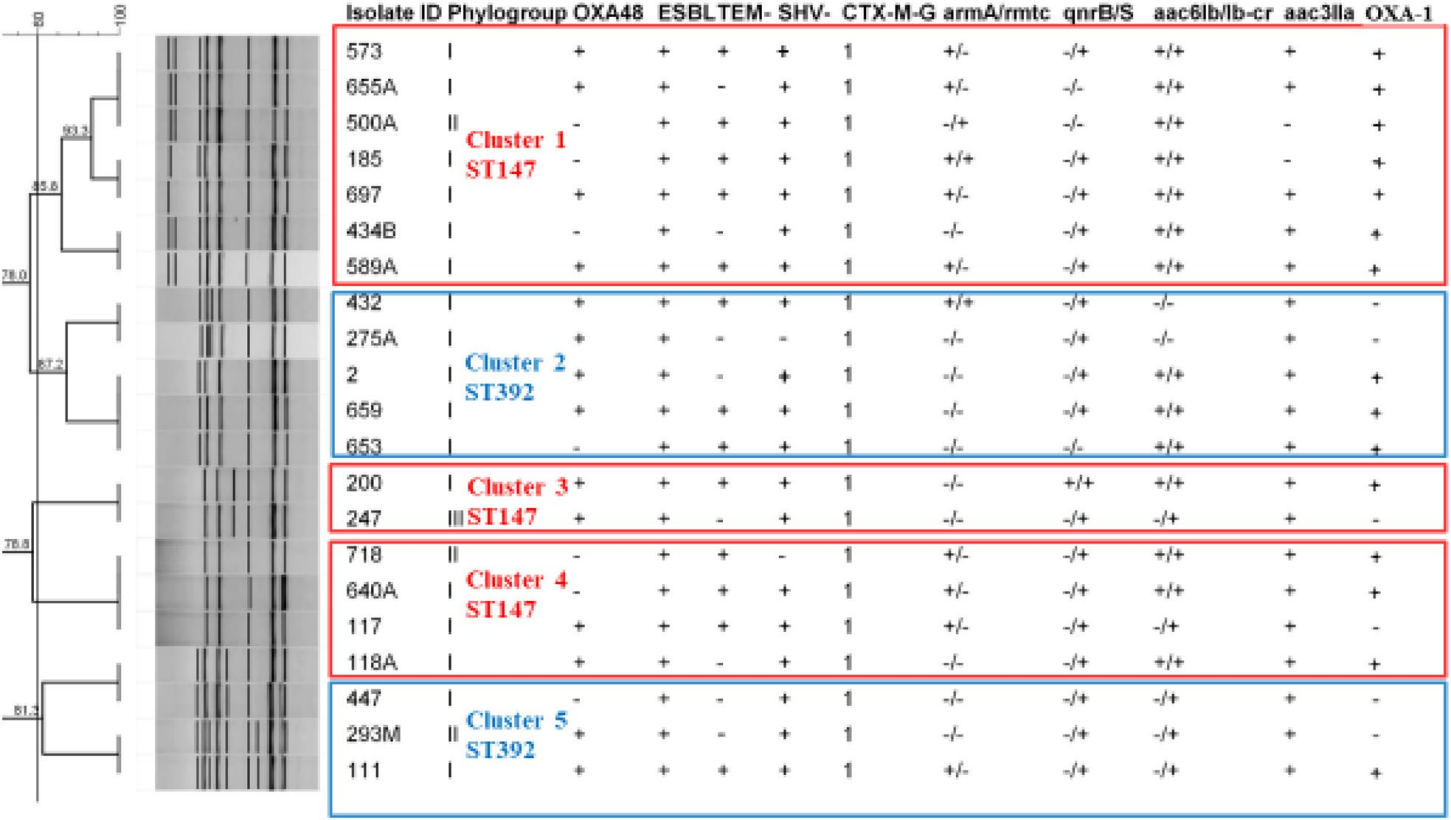
ERIC-PCR based dendogram of *bla*_NDM_ producers. Five representatives were subjected to MLST and two STs, including ST147 and ST392 were identified. ESBL: indicate the phenotypic results

Based on the ERIC-fingerprinting of *bla*_NDM_ negative isolates, 25 clusters were detected, including of two to 10 isolates in each cluster. Twenty-six isolates were also found as singletons (Fig. 2). In contrast, NDM carrying strains were grouped into five clusters (Fig. 3). Consequently, as the representatives of NDM producers, one strain was selected from each of the cluster and subjected to MLST assay. Two sequence types, ST392 (n = 9) and ST147 (n = 12) were identified among NDM positive isolates. Noted that the ST392 is the clonal complex (CC) of ST147. The first *bla*_NDM-1_ strain was obtained on March 2015 from internal intensive care unit (ICU) followed by the couple of isolates retrieved on May from surgical ICU. On June, there was only one strain isolated from a patient hospitalized in internal ward. On July, we obtained one *bla*_NDM-1_ isolate from each of the internal ICU and surgical ICU sites. On August, there were three isolates; two from internal ICU and one from internal ward. On the following months from October 2015 to February 2016, nine strains were isolated from patients hospitalized at internal ICU. On March, we obtained one isolate from internal ward and afterward we received two other isolates from coronary care unit (CCU) and surgery wards on April. The last positive strain recovered from a dialysis patient whom was initially hospitalized in internal ICU on September 2016.

### Phylogroups, capsular genotypes and hypervirulent clones

Except for *Klebsiella oxytoca* strain (strain no. 511b) which showed a different RFLP-pattern, all the remaining 121 studied isolates were grouped in three phylogroups, including 98 (80.3%) in group I, 20 (16.4%) in group II and three (2.5%) in group III. Most of the NDM-1 producing isolates, including 17 (81%) strains, belonged to the group I, and the remaining four strains belonged to group II and III. Of the CTX-M genes, the CTX-M-G2, G8 and G9 were detected exclusively among isolates of phylogroup I. Of the six capsular types studied; K1 and K2 genotypes were identified among one and 15 isolates, respectively. The K1 positive strain was cultured from urine specimen, while 12 (80%) and three (20%) of K2 strains were recovered from urine and sputum cultures, respectively. Carbapenem resistant and MDR phenotypes were detected among five (33.3%) and 11 (73.3%) K2 positive strains, respectively. The K1 strain detected in phylogroup I, while the K2 isolates belonged to both phylogroup I (14 [93.3%]) and II (1 [6.7%]) (*P *> 0.05).

Based on the carriage of *peg344*, *iucA*, *iroB*, *rmpA* and *rmpA2* biomarkers, three strains, including K1 (strain 542, carbapenem susceptible) and two K2 (strain 290, carbapenem resistant and 533, carbapenem susceptible) isolates were identified as hvKp. Virulence genes *iutA*, *mrkD* and *ybtS* were detected in these three hvKp strains, except for K1 isolate which was negative for the latter gene. The two K2^+^/hvkp strains (strains 533 and 290) were identified as ST86 using the hvKP-K2 multiplex PCR. As expected, this multiplex PCR was completely negative for the only K2^+^/phylogroup II strain (strain 608). The *alls* gene was not detected among study isolates. None of the *bla*_NDM-1_ producers were positive for K1/K2 capsular types and were not identified as hvkp.

We also studied the presence of virulence genes in all carbapenem resistant isolates and *peg344*, *iucA*, *rmpA*, *rmpA2*, *iroB1*, *iroB2, iutA*, *mrkD* and *ybtS* were detected in 1.8%, 1.8%, 1.8%, 1.8%, 7.3%, 12.7%, 18.2%, 89.1% and 67.3%, of strains, respectively. Higher rates of *iutA* and *ybtS* carriage were detected among *bla*_NDM-1_ positive (19% and 71.4%) strains as compared to negative (17.6% and 64.7%) ones, while the differences were not statistically significant (*P *> 0.05). Of the resistance genes studied, the carriage of *qnrB* and *qnrS* had positive and negative association with K2 capsular type, respectively.

## Discussion

The first report of NDM producing Gram–negative bacilli from Iran was described in 2013 in a *K. pneumonia*e strain, which was cultured from the urine specimen of a patient with kidney transplant rejection history [23]. In the current study, we investigated the molecular epidemiology of a group of *bla*_NDM-1_ producing isolates cultured from various extraintestinal specimens collected from Kosar hospital, Semnan, Iran. In our study, NDM-1 producing *K. pneumoniae* strains were mostly derived from urine specimens, followed by sputum of patients admitted to different hospital wards.

In our study and comparison to NDM negative isolates, *bla*_NDM-1_ carrying strains were much resistant to different classes of β-lactam and non β-lactam antibiotics by disk diffusion assay. It has been shown that tigecycline, fosfomycin and colistin are the most potent agents against NDM producing isolates [8]. While we didn’t determine the susceptibility patterns of NDM producing strains against these antibiotics, amikacin which found with the highest susceptibility rates may be considered as a treatment option due to some limitation of the extensive clinical usage of the three aforementioned antibiotics.

Phylogrouping of study *Klebsiella* isolates identified three groups, with the phylogroup I as the dominant group, followed by group II and III. It has been shown that the level of resistance for most of the antibiotics is highest among phylogroup I, intermediate in group II and lowest in group III, with the highest number of normal microbiota among the latter group [24]. Accordingly, most of the *bla*_NDM-1_ producing strains (81%) belonged to group I and co-harbored different resistance genes, including *bla*_SHV-_, CTX-M-G1, *qnrS*, *aac6*-*Ib*-*cr* and *aac3IIa*. While the resistance is a critical parameter for the transmission of phylogroup I *K. pneumonia*e strains, detection of NDM/OXA-48 carbapenemases and consequently carbapenem resistant phenotype among phylogroup II (strains: 500A, 718, 293 M) and III (isolate: 247) strains, confirm the role of this mainly nosocomial and opportunistic pathogen in the dissemination of resistance elements.

Among the *bla*_NDM-1_ producers, 11 (52.3%) strains co-harboured any β-lactamase gene, CTX-M; any PMQR; and 16S rRNA methylase gene *armA* or *rmtC*. PMQR coexisting with any β-lactamase gene was also high (21 [100%]). A strong association between the carriage of NDM and 16S rRNA methylase encoding gene, specifically *rmtC* methylase has been shown [25]. While the prevalence of *rmtC* among *bla*_NDM-1_ producing strains was higher than the negative ones, this association was found borderline. In contrast, the co-occurrence of *armA* or the other aminoglycoside resistance genes with NDM was found significant. So our results indicate that NDM producing *Klebsiella* isolates, have acquired a broad spectrum of singular and different resistance determinants.

It has been reported that carbapenemase producing *K. pneumoniae* strains have a different population and less genetic diversity as compared to carbapenem susceptible isolates [26]. In our study, ERIC-PCR showed five clusters and higher genetic homogeneity among *bla*_NDM-1_ producers as compared to negative ones. Accordingly, the MLST assay identified two STs among NDM producing isolates. ST392, which is an SLV of ST147, has been associated with the carriage of *bla*_KPC_, *bla*_NDM_ and *bla*_OXA-48_ [27]. In the current study, ST392 has detected among NDM positive isolates with different ERIC patterns. Another detected ST, ST147 which is an international clone, has been reported from center, south and south east of Iran [6–8]. In our study, ST147 has been associated with CTX-M-G1, *bla*_SHV-_, *aac6Ib*-*cr* and *armA*. This ST has been recognized as a pandemic clone and is considered as a threat to public health worldwide [28]. ST147 was firstly observed by Papagiannitsis et al., as hosting the *bla*_VIM_ gene, and then Samuelson et al. reported this clone among *K. pneumonia*e isolates imported to Scandinavia, mostly from Greece [28]. So, these findings imply that ST147 has the potential to acquire different resistance elements, of note, *bla*_NDM/OXA-48_ carbapenemases and facilitate their rapid spread into the other pandemic clones of *K. pneumoniae*.

String test, a phenotypic assay that is commonly used to identify the hvKp strains, has been shown to performed suboptimally, particularly in low prevalence areas [18]. So, the identification of some genetic markers including *peg344*, *irob*, *rmpA*, *rmpA2* and *iucA* has been suggested for accurate differentiation between hvKp and classical *K. pneumoniae* strains [18]. Accordingly, one K1 and two K2 ST86 strains were identified as hvKp, of those one ST86 strain was MDR and carbapenem resistant. While these capsular types have not generally been associated with acquired resistance genes at the time which were identified, in the last few years increasing reports of resistant strains were observed among these genotypes [29]. In agreement with these new reports, four out of five carbapenem resistant K2 strains harboured the *bla*_OXA-48_ gene. It has been reported by Turton et al., that isolates of CC147 carrying *bla*_OXA-48_ or *bla*_NDM_ which was resistant to colistin, harboured many antibiotic resistance determinants, and contained a quarter of the virulence genes which were found in the K1-ST23/OXA-48^+^ isolates [30]. While our CC147 strains (all carrying NDM) were K1/K2/hvkp negative, they harboured relatively higher rates of siderophores *iutA* and *ybtS* as compared to another carbapenem resistant NDM negative isolates. So, concerning this finding that the acquisition of any one of the siderophore clusters increases the risk of complicated infections [2, 30], the combination of virulence and antibiotic resistance in this pandemic clone is extremely worrying.

Our study had some limitations. The *bla*_OXA-48_ producers and K1/K2 strains were not subjected to sequence type determination. Furthermore, the TEM- and SHV- variants of positive isolates were not determined. We investigated the carriage of some limited virulence factors among carbapenem resistant strains, while the other important virulence determinants remained to be characterized among both of carbapenem susceptible and resistant isolates.

## Conclusions

In summary, clonal dissemination of *bla*_NDM-1_ carrying *K. pneumoniae* that co-harbour different β-lactamases, aminoglycoside modifying enzymes, and PMQR determinants have been observed. Isolation of carbapenem resistant *K. pneumonia*e strains from clinical sources has been reported from Iran, previously. However, their association with K1 or K2 hypervirulent capsular types has never been reported. The emergence of the CC147 carbapenem resistant *K. pneumoniae* strains warrants urgent surveillance because not only are they considered as international clone, but also they simultaneously have higher rates of siderophores in a pandrug resistance profile.

## Data Availability

The data can be accessible to the interested researchers by the corresponding author on behalf of all authors on reasonable request.
